# Editorial: RNAs at the crossroads between effectors and targets; discovery and development of new drugs

**DOI:** 10.3389/fmolb.2023.1157440

**Published:** 2023-02-27

**Authors:** Federica Chiappori, Luca Mollica

**Affiliations:** ^1^ National Research Council—Institute for Biomedical Technologies, Milan, Italy; ^2^ L.I.T.A, Department of Medical Biotechnologies and Translational Medicine, University of Milan, Milan, Italy

**Keywords:** RNA drug target, RNA as drug, cancer, miRNA, aptamer, RNA structure

In recent years, several functions of different RNA molecules have been identified, as well as the ability of these molecules to regulate multiple processes in the cell, allowing the identification of RNA molecules as a large class of promising novel drug targets. Moreover, some small RNA molecules were recently approved in therapy or are being developed, because of their efficacy in treating diseases untreatable with classic drugs that target proteins; such as diseases caused by alternative spicing. Besides, small RNAs have the possibility to rapidly personalize treatments. Since 1998, eight Antisense oligonucleotides (ASO), four small interfering RNA (siRNA), one aptamer, and two messenger RNA (mRNA) vaccines were approved by the U.S. Food and Drug Administration (FDA) (Kim, 2022). These demonstrate their efficacy in treating several diseases such as Duchene Molecular Dystrophy, amyloidosis, and hypercholesterolemia.

To analyse available experimental and computational methods and recent developments in the therapeutic RNA field, we have collected five contributions to the present Research Topic: Three original research articles and two review articles.

Despite great advances, ranging from surgery to pharmaceutical sciences, it remains a challenge both to understand the development mechanisms of cancer and to identify specific and successful treatments for the disease. Four out of the five contributions to this Research Topic explore cancer from different perspectives, all of them focussing on the crucial role of RNA interactions at several levels.

The review proposed by Yi and Yu discusses the regulation of Solute Carrier (SLC) superfamily transporters expression by microRNA molecules (miRNAs). This transporter family is responsible for nutrients and drug-dysregulated transport in cancer cells. Moreover, several miRNAs were found to regulate SLC expression. For these reasons, miRNA-mediated regulation of SLC transporters in cancer therapy can override chemoresistance and/or help therapies to prevent cancer cells from “feeding.”

Yu et al. report the development of the Prediction of SM-miRNA Regulation pairs (PSRR) web server. The inhibition of miRNA by small molecules can be useful to regulate cellular processes acting on a single target. miRNAs can be targeted by small molecules. To identify miRNA targets for cancer therapies, the authors implement a web server that predicts the regulation relations between miRNAs and small molecules. Different predicting algorithms were implemented, but the random forest returns the best performances.

The work of Esposito et al. group describes a modified cell-SELEX approach for the isolation of RNA aptamers specific for hypoxia-related epitopes expressed on breast cancer cell surfaces. Owing to the central role of hypoxia in tumor progression, treatment failure, and negative prognosis, the authors developed a “Hypoxic cell-SELEX” methodology that relies on a simple and rapid system to reproduce *in vitro* the hypoxic conditions present in the tumor micro-environment. In this context, they report the identification and the biophysical characterization of the most represented aptamer with improved binding affinity for the hypoxic phenotype.

Cheng et al. propose an innovative nanobubble complex to be used in the sonodynamic treatment of Hepatocellular carcinoma. The treatment induces cellular apoptosis. The authors also evaluate the differentially expressed RNA (mRNAs, lncRNAs, and circRNAs) before and after treatment, and a subset of differentially expressed genes as potential targets for HCC treatment.

Finally, the recent advancements in experimental resolution and the *ad hoc* computational modeling pipelines for RNA structures have enabled the growth of RNA-based therapy. In this context, Mollica et al. summarize the recent advances and applications of therapeutic RNAs, and the structural viewpoint, the available strategies, and the computational and experimental analysis methods with a focus on dynamic and flexibility aspects and binding analysis.

Overall, the contributions highlight the interest in focussing on RNA both as a target and as a treatment, and the possibility of combining experimental and computational methods to increase the efficacy of the analysis. Besides, we believe that the study of RNA structures in conjunction with a biological and pharmacological characterization of RNA interactors and interactions could be an asset for future development in the field. The RNA structure is still not often considered in the design of new therapies, but some recent research emphasizes this aspect (Duchardt-Ferner et al., 2020; Mitchell et al., 2020; Li et al., 2021). We believe this Research Topic will be useful for biotechnologists and biologists, as well as for computational and biophysical chemists willing to drive their research in a still slightly immature but very promising field.

We thank all Authors and Reviewers for their contributions to this Research Topic and we acknowledge the Frontiers in Molecular Biosciences Team for its support.
